# On the Mutual Relationships between Molecular Probe Mobility and Free Volume and Polymer Dynamics in Organic Glass Formers: *cis*-1,4-poly(isoprene)

**DOI:** 10.3390/polym13020294

**Published:** 2021-01-18

**Authors:** Helena Švajdlenková, Ondrej Šauša, Sergey V. Adichtchev, Nikolay V. Surovtsev, Vladimir N. Novikov, Josef Bartoš

**Affiliations:** 1 Polymer Institute of SAS, Dúbravská Cesta 9, 84541 Bratislava, Slovakia; jozef.bartos@savba.sk; 2 Institute of Physics of SAS, Dúbravská Cesta 9, 84511 Bratislava, Slovakia; ondrej.sausa@savba.sk; 3 Department of Nuclear Chemistry, Faculty of Natural Sciences, Comenius University, Mlynska Dolina, Ilkovicova 6, 84215 Bratislava, Slovakia; 4 IA&E, Russian Academy of Sciences, 630090 Novosibirsk, Russia; adich@ngs.ru (S.V.A.); saa@iae.nsk.su (N.V.S.); novikov@iae.nsk.su (V.N.N.); 5 Novosibirsk State University, 630090 Novosibirsk, Russia

**Keywords:** ESR, TEMPO, *cis*-1,4-poly(isoprene), PALS, LS

## Abstract

We report on the reorientation dynamics of small spin probe 2,2,6,6-tetramethylpiperidinyl-1-oxyl (TEMPO) in *cis*-1,4-poly(isoprene) (*cis*-1,4-PIP10k) from electron spin resonance (ESR) and the free volume of *cis*-1,4-PIP10k from positron annihilation lifetime spectroscopy (PALS) in relation to the high-frequency relaxations of *cis*-1,4-PIP10k using light scattering (LS) as well as to the slow and fast processes from broadband dielectric spectroscopy (BDS) and neutron scattering (NS). The hyperfine coupling constant, 2*A_zz_*′(*T*), and the correlation times, *τ*_c_(*T*), of *cis*-1,4-PIP10k/TEMPO system as a function of temperature exhibit several regions of the distinct spin probe TEMPO dynamics over a wide temperature range from 100 K up to 350 K. The characteristic ESR temperatures of changes in the spin probe dynamics in *cis*-1,4-PIP10k/TEMPO system are closely related to the characteristic PALS ones reflecting changes in the free volume expansion from PALS measurement. Finally, the time scales of the slow and fast dynamics of TEMPO in *cis*-1,4-PIP10k are compared with all of the six known slow and fast relaxation modes from BDS, LS and NS techniques with the aim to discuss the controlling factors of the spin probe reorientation mobility in polymer, oligomer and small molecular organic glass-formers.

## 1. Introduction

The structural and dynamic origin of the vitrification i.e., a transition of a glass-forming material from its normal liquid state through the supercooled liquid one into the glassy one is a long-term investigated phenomenon [1,2,3,4]. The structure and dynamics of material can be study via traditional-direct methods such as X-ray and neutron diffraction [5,6], neutron and light scattering [7,8,9,10], nuclear magnetic resonance (NMR) [11] and broadband dielectric spectroscopy (BDS) [12,13].

The main findings in the dynamic behavior of glass-forming organics studied by BDS, including oligo- and polymers, are the deviations of a structural relaxation from the ideal exponential behavior in the time dependence of the relaxation response, the so-called non-Debye behavior, as well as in the temperature dependence of the characteristic time scale of the structural α relaxation, *τ_α_*, the so-called non-Arrhenius behavior [1,2,3,4].

The dynamic changes in dipole reorientation given by the cross-over bends in the temperature and time dependences are marked by the characteristic dynamic temperatures *T*^DYN^, such as the Arrhenius temperature, *T*_A_, in viscosity or BDS data [14,15] and the Stickel temperature, *T*_B_^ST^ [16], the Schönhals temperature, *T*_B_^SCH^ [17], as well as the Alegría–Colmenero–Ngai temperature, *T*_B_^ACN^ = *T*_B_^βKWW^ [18,19,20], from BDS measurements. In addition, both slowdown of the structural dynamics accompanied by a broadening of the distribution of relaxation times reflex a development of the dynamic heterogeneity of the glass-formers [1,2,3,4]. These aspects are responsible for the afore-mentioned deviations of the large-scale structural relaxation and the small-scale local secondary relaxations [21]. In addition to the mentioned phenomenological features, there exists only a few theoretical works with inclusion of dynamic heterogeneity, such as recently published the generalized Adam–Gibbs model [22].

The structural-dynamic evolution of glass-form can be also investigated through the extrinsic probes, i.e., an atomic *ortho*-positronium probe (*o*-Ps) by positron annihilation lifetime spectroscopy (PALS) [23,24] and stable molecular nitroxide radicals via electron paramagnetic resonance (ESR) [25,26,27]. These external probes are highly sensitive to the *local* structural-dynamic changes over a broad temperature range. The PALS technique detects the annihilation behavior of *o*-Ps probe through the *o*-Ps lifetime, *τ*_3_(*T*), reflecting the local free volume changes in a given glass-former over a wide temperature range. In ESR, the rotational dynamics of spin probes, such as 2,2,6,6-tetramethyl-piperidinyl-1-oxy (TEMPO), within a diamagnetic glass-former is observed in the ESR spectra. They can be evaluated via two dynamic parameters, the spectral parameter of the probe mobility, 2*A*_zz′_(*T*), and the correlation times, *τ*_c_(*T*). In both extrinsic probe techniques, the corresponding PALS and ESR temperature dependences of various glass formers are highly non-monotonic. They exhibit several regions of the different thermal behavior of both probes and their change as are marked by the characteristic PALS temperatures *T*_bi_^PALS^ of various bend effects [28] and the characteristic ESR ones of distinct crossover effects: *T*_50G_ and *T*_Xi_^ESR^ [29,30]. Their mutual coincidences indicate that the changes in the *τ_3_* and 2*A*_zz′_ quantities with temperatures are controlled or, at least, influenced by the same physical process [31,32].

Moreover, the comparison between the characteristic PALS temperatures with afore-mentioned dynamic characteristic temperatures, *T*^DYN^, and the dynamic time scales, the two bend effects in the PALS response above the glass-to-liquid transition at *T*_b2_^L^ and *T*_b1_^L^ are related to the structural α-relaxation or sometimes, to the secondary β-process [31,32,33,34,35,36]. On the other hand, concerning the characteristic ESR temperatures, their mutual relationships with dynamic ones appears to be more complicated. However, for some glass formers, the characteristic electron spin resonance (ESR) and dynamic (DYN) temperatures in temperature dependences mutually coincide [31,32] The full interpretation of the spin probe dynamics in a given medium requires to reveal the origin of temperature coincidence(s), i.e., the mutual relationship between relaxation dynamics of the pure substance and the spin probe reorientation (the dynamic response of the spin probe system substance/spin probe) [37,38].

Recently, a combined ESR, PALS and BDS work on oligomer *cis*-1,4-poly(isoprene) (*cis*-1,4-PIP0.8k) consisting of twelve monomer units per chain was reported [39]. Here, in the low-T and high-T regions, the unimodal broad or narrow line shape of ESR spectra reflect the slow or fast motion regime of spin probe TEMPO, respectively. In the intermediate-*T* region, the bimodal EPR spectra imply the dynamic heterogeneity of the spin probe TEMPO. In the comparison of ESR and PALS responses, several coincidences between the acceleration of the spin probe dynamics at *T*_xi_ and the free volume expansion at *T*_bi_ were revealed. Finally, some of these changes were tentatively ascribed to certain features of the structural α relaxation [25,26,27]. In spite of the numerous temperature coincidences, the time scales of spin probe reorientation and relaxation dynamics of given matrix are not in agreement. Even at high temperatures, the reorientation time scale of molecular probe is shorter than that of the segmental α process.

On the other hand, for a few small molecular glass formers, such as *n*-propanol [38] or glycerol [37], the full coupling between the time scales of the spin probe reorientation in the fast motion regime and the reorientation of medium constituents was found. One possible explanation for a decoupling of time scales found in *cis*-1,4-PIP0.8k may be that some other faster motional process(es) controlling the spin probe reorientation could be detected by an appropriate high-frequency dynamic technique.

Very recently, a preliminary joint ESR and PALS study of polymer *cis*-1,4-PIP10k revealed the dynamic heterogeneity of the spin probe TEMPO, i.e., slow and fast component in the supercooled state of polymer as well as mutual coincidences between ESR and PALS temperatures [40].

The aim of this investigation is (i) to carry out a detailed joint study of the polymeric *cis*-1,4-poly(isoprene) (*cis*-1,4-PIP10k) sample with the higher molecular weight, above the entanglement situation, by PALS, ESR and suitable high-frequency dynamic techniques, namely, broadband dielectric spectroscopy (BDS) and especially, light scattering (LS) (ii) to compare the ESR and PALS findings of oligomeric and polymeric *cis*-1,4-poly(isoprene) (*cis*-1,4-PIP0.8k vs. *cis*-1,4-PIP10k) and finally, (iii) to discuss a role of the free volume from PALS and the fast motional modes obtained from LS and BDS data and to clarify the controlling factors of spin probe reorientation by combining the ESR data with other high-frequency dynamic results from BDS and detailed LS measurements.

## 2. Materials and Methods

### 2.1. Materials

The studied polymeric glass former was *cis*-1,4-poly(isoprene) (*cis*-1,4-PIP10k) from Scientific Polymer Products, Inc. with the number average molecular weight *M*_n_ = 9550 g/mol, *PDI* ≈ 1.03 and *T*_g_^DSC^ = 208 K. In ESR experiments, 2,2,6,6-tetramethyl-1-piperidinyloxy (TEMPO) from Sigma Aldrich, Inc., Germany was used as a spin probe. TEMPO was dissolved in *cis*-1,4-PIP10k medium at a very low concentration of approximately 5 × 10^−4^ M.

### 2.2. Electron Spin Resonance (ESR)

ESR measurement of *cis*-1,4-PIP10k/TEMPO solution was carried out on the X-band Bruker-ER 200 SRL (Stuttgart, Germany) at constant frequency 9.4 GHz, with a manual control of the heater and the gas flow (Bruker BVT 100). ESR spectra of *cis*-1,4-PIP10k/TEMPO system were recorded during heating over a wide temperature range from 100 K to 350 K. The temperature stability was ±0.5 K. The sample at each temperature was kept for 15–20 min before starting to accumulate two ESR spectra. The ESR lineshape analysis was in terms of the spectral parameter of 2*A*_zz′_(*T*) [25,26,27] and the correlation times in the slow and fast motion regimes, *τ*_c_^slow^(*T*),*τ*_c_^fast^(*T*) and the corresponding fractions of the spin probes in the respective motion regime *F*^slow^(*T*), *F*^fast^(*T*) [41].

### 2.3. Positron Annihilation Lifetime Spectroscopy (PALS)

PALS lifetime spectra were obtained by the conventional fast-fast coincidence spectrometer using plastic scintillators coupled to Philips XP 2020 photo-multipliers, Photonis S.A.S., Brive, France. The time resolution of spectrometer was about 320 ps. The *cis*-1,4-PIP10k sample was measured under vacuum over a wide temperature range from 100 K to 350 K by helium closed-cycle refrigerator Janis CCS-450, Lake Shore Comp., Woburn, MA, USA. The temperature stability was around 1 K. The measuring time per one spectrum at each temperature was at least 2 h. The LifeTime (LT) program (version LT polymers) [42] was applied for the analysis of lifetime spectra into three components. Here, the relative contribution of *para*-positronium (*p*-Ps) and *ortho*-positronium (*o*-Ps) annihilation to lifetime spectra was 1:3. A short lifetime component from *para*-positronium annihilation (*p*-Ps) was fixed at *τ*_1_ = 0.125 ns, an intermediate one *τ*_2_ was attributed to the annihilation of free positrons e^+^ in bulk, small free volumes and defects. Finally, a long component *τ*_3_ originates from the *ortho*-positronium (*o*-Ps) annihilation. The *o*-Ps probe annihilates in organic materials via a pick-off process in which e^+^ from the *o*-Ps annihilates with the e^−^ of cavity surface, while the *o*-Ps lifetime is shortened, depending on the size and shape of cavity [43,44,45,46,47,48,49].

### 2.4. Broadband Dielectric Spectroscopy

The BDS spectra of *cis*-1,4-PIP10k were measured by Concept 80 Novocontrol spectrometer (Novocontrol Technologies GmbH & Co., Montabaur, Germany) over a wide frequency range, 10^−2^–10^8^ Hz, and the temperature range from 190 K to 385 K. The results were already reported in Ref. [50]. Briefly, the temperature evolution of BDS spectra for *cis*-1,4-PIP10k exhibits the normal, i.e., chain (n-) mode and the primary, i.e., segmental (α-) relaxation one at lower or higher frequencies, respectively. The characteristic relaxation times of both the relaxation modes, *τ*_α_(*T*), *τ*_n_(*T*), were obtained by applying two Havriliak–Negami (HN) functions [51,52].

### 2.5. Light Scattering 

Depolarized light scattering spectra of the pure *cis*-1,4-PIP10k sample were measured in a back-scattering geometry using the tandem Fabry-Perot interferometer (Sandercock model, Ottawa, ON, Canada) at frequencies below ~300 GHz and the Raman spectrometer (Trivista 777, Teledyne Princeton Instruments, Trenton, NJ, USA) at frequencies above~100 GHz.

The experiment with Raman spectrometer was performed in a right-angle scattering geometry by using a solid-state laser with a wavelength of 532 nm (Millennia, Spectra Physics, Santa Clara, CA, USA). An additional monochromator was used to suppress the spurious secondary laser lines in the excitation beam [53]. The spectral resolution of the spectrometer was about 30 GHz. Wavelength calibration of the spectrometer was done by comparing of the measured spectrum of a neon discharge lamp and tabular data. The *cis*-1,4-PIP10k sample was sealed in a cylindrical glass cuvette. For relatively low-temperature measurements (200–320 K), the sample was attached to a cold finger of an optical closed-cycle helium cryostat (Advanced Research Systems, Inc., Macungie, PA, USA) through an indium gasket. In the case of high-temperature measurements (330–380 K), the sample was placed in a home-built oven.

The experiment with tandem Fabry–Perot interferometer (TFPI) was carried out in a back scattering geometry by using a solid-state laser operating at the wavelength of 532 nm (Excelsior, Spectra Physics, Santa Clara, CA, USA). For performed measurements at different temperatures, a hermetically sealed flat cuvette with *cis*-1,4-PIP10k was placed inside of a liquid nitrogen cryostat (Linkam, Scientific Instruments, Tadworth, UK). In order to cover a wide frequency range, the spectra at four free spectral ranges 5 GHz, 15 GHz, 75 GHz, 300 GHz were measured. The narrow interference filter was used to suppress higher transmission orders of the interferometer [54,55]. For an accurate evaluation of the spectral shape, all spectra were corrected on the transmission function of the interferometer as described in Ref. [56]. In the end, the spectra measured with TFPI were combined with those obtained by Raman spectrometer.

In LS technique, light scattering susceptibility spectra χ”(ν) of *cis*-1,4-PIP10k were collected at selected temperatures from the *T* range: 200–380 K. Based on the dynamic susceptibility, the light scattering data are able to directly compare to dielectric loss (ε″) obtained from broadband dielectric spectroscopy (BDS). The spectra consist of the tail of the segmental relaxation and the fast dynamics which appears above 30 GHz. Light scattering data extend the segmental relaxation outside of the frequency window accessible to the broadband dielectric spectroscopy (BDS) [50].

## 3. Results

### 3.1. ESR Data

**Figure 1:**
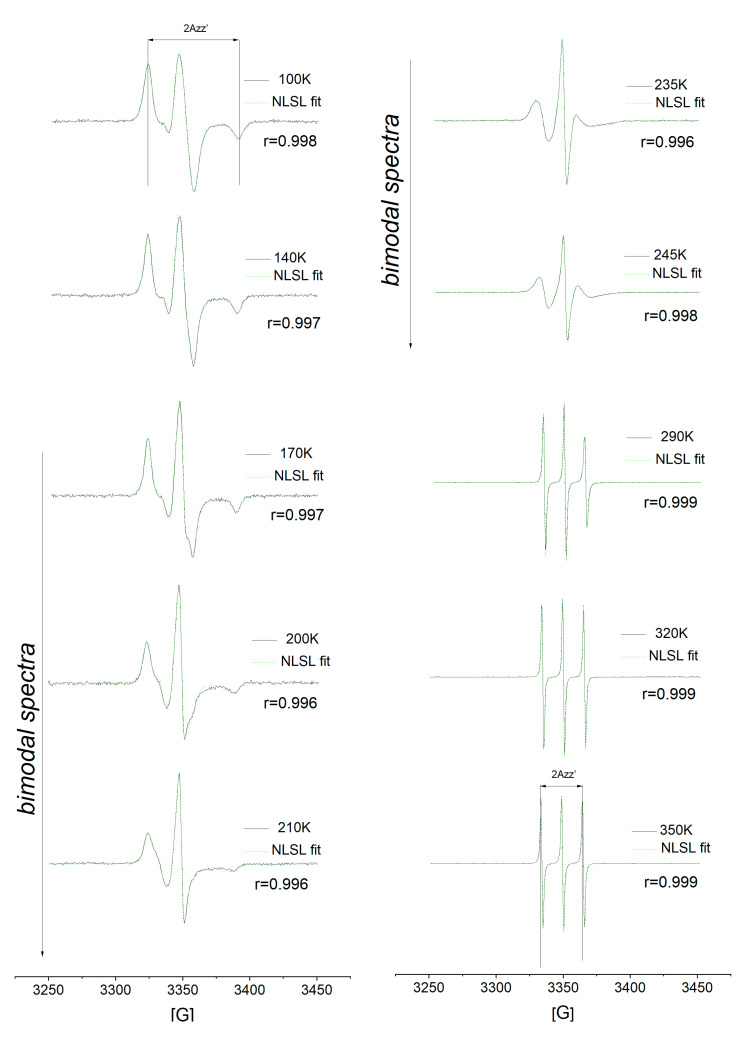
Spectral evolution of the spin system of *cis*-1,4-poly(isoprene) and TEMPO (*cis*-1,4-PIP10k/TEMPO) at selected temperatures over the temperature range from 100 K to 350 K. The experimental data (black) and simulated (green) electron spin resonance (ESR) spectra of *cis*-1,4-PIP10k/TEMPO system within the slow motion regime at 100 K, 140 K, superimposed slow and fast motion regimes at 170 K up to 245 K and within the fast motion regime at 290 K up to 350 K are shown. The distance of the outer line separation, 2*A*_zz′_ displays the double-sided arrow.

Figure 1 shows the spectral evolution of the spin system *cis*-1,4-PIP10k/TEMPO over a broad temperature range from 100 K to 350 K. The ESR spectra reflect the changes in reorientation dynamics of TEMPO probe from the broad anisotropic spectra of slow spin probe motion at low temperatures through the bimodal spectra to the narrow triplet spectra of fast spin probe reorientation in the high *T* region. The quite wide region of the bimodal spectra occurred in the temperature range from ca. 155 K up to ca. 245 K, where the slow- and fast-moving spin probes are superposed.

As mentioned in the Experimental section, the obtained ESR spectra can be evaluated in terms of the distance of the outer line separation, 2*A*_zz′_ [25,26,27] and the correlation times, *τ*_c_^slow^(*T*),*τ*_c_^fast^(*T*), as well as their related population fractions, *F*^slow^(*T*), *F*^fast^(*T*) [41].

#### 3.1.1. Spectral Parameter of Mobility, 2A_zz′_

**Figure 2:**
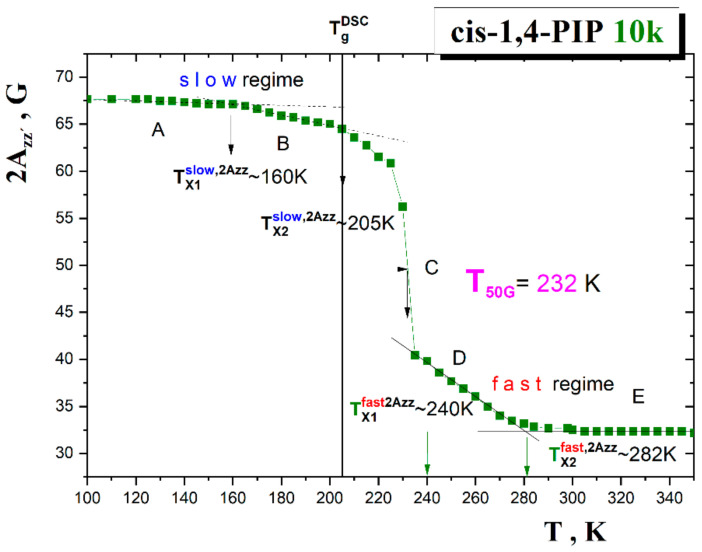
The distance of the outer line separation of spectra, 2*A*_zz′_, as a function of temperature for TEMPO mobility in *cis*-1,4-PIP10k. Two weak decreases at *T*_X1_^slow,2Azz~^160 K and *T*_X2_^slow,2Azz~^205 K within the slow motion regime followed by the main transition of spin probe reorientation from the slow to fast motion regime at *T*_50G_ = 232 K and finally, by the onset and crossover effects within the fast regime at *T*_X1_^fast,2Azz~^240 K and *T*_X2_^fast,2Azz~^282 K occur. The glass-to-liquid transition temperature *T*_g_^DSC^ is also included by vertical line.

Figure 2 shows the temperature dependence of the distance of the outer line separation, 2*A*_zz′_ of spectra of TEMPO/cis-1,4-PIP10k system over a wide temperature range from 100 K to 350 K. Five regions of distinct thermal behavior of 2*A*_zz′_ reflecting different spin probe mobility can be distinguished. They are marked as A–E.

At the lowest temperatures, the broad spectrum reaches the extrema separation value of 2*A*_zz′_ (100 K) = 67.5 ± 0.2 Gauss which lies in the typical range for van der Waals organic compounds [32].

In the low temperature regions A and B of the slow motion regime, the 2*A*_zz′_ parameter of mobility starts to decrease slightly at the first characteristic ESR temperature *T*_X1_^slow,2Azz^ ≅ 160 K and next, at *T*_X2_^slow,2Azz^ ≅ 205 K. The second value is situated in the vicinity of the glass-to-liquid temperature of *cis*-1,4-PIP10k *T*_g_^DSC^ = 208 K as detected by DSC technique [50]. This relative closeness of the *T*_X2_^slow,2Azz^ and *T*_g_^DSC^ values indicates that the second change, i.e., acceleration in the spin probe TEMPO mobility during heating is closely related to the glass-to-supercooled liquid transition as the basic thermodynamic signature and a descriptor of any amorphous materials.

The most pronounced effect in the 2*A*_zz_-*T* plot is a transition of the spin probe TEMPO reorientation from the slow to fast motion regime within the region C which is usually quantified operationally by the characteristic ESR temperature *T*_50G_. At this temperature, the outermost peaks separation in the triplet signal reaches just 50 Gauss. Its value for the *cis*-1,4-PIP10k/TEMPO system is *T*_50G_ = 232 K.

Finally, within the fast regime of the spin probe TEMPO reorientation, two regions D and E are observed which are marked by the characteristic ESR temperature, i.e., an onset to the fast regime at *T*_X1_^fast,2Azz^~240 K and further acceleration of TEMPO mobility at *T*_X2_^fast,2Azz^ lying at around 282 K. This simple measure of the spin probe mobility in relation to free volume findings and the origin of all characteristic ESR temperatures will be discussed later.

#### 3.1.2. Spectral Simulations

The rotational dynamics of TEMPO in *cis*-1,4-PIP10k can be also evaluated by the more sophistic approach via spectral simulations. The advantageous program is represented by the Non-linear Least Squares Line (NLSL) code based on the isotropic Brownian model of the spin probe reorientation and provides the time scales of slow and fast moving components *τ*_c_^slow^(*T*), *τ*_c_^fast^(*T*) as well as their population fractions *F*^slow^(*T*), *F*^fast^(*T*) [41].

Figure 1 represents a comparison between the experimental and simulated one- or two-component ESR spectra over a wide temperature range. The unimodal ESR spectra were found in the low-*T* range from 100 K to 150 K as well as in the high-*T* one from 250 K to 350 K. Bimodal ESR spectra from the superimposed slow and fast components were appeared in the intermediate-*T* range between 155 K and 245 K as documented by the good quality of fits ranging between 0.994 and 0.998.

**Figure 3:**
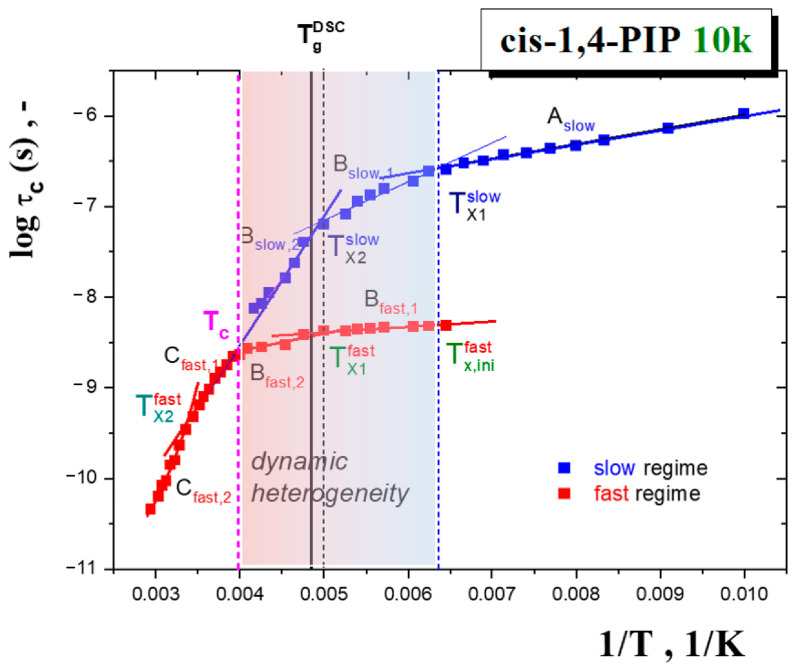
Arrhenius plot of the correlation time of TEMPO probe in *cis*-1,4-PIP10k medium exhibits two unimodal regions, i.e., low-*T* region A and high-T region C and one superimposed bimodal region B with the dynamic heterogeneity of the spin probe TEMPO mobility from *T*_X1_^slow^ = *T*_X,ini_^fast^~155 K, to *T*_c_~250 K [40]. The second acceleration of TEMPO within slow regime at *T*_X2_^slow^~203 K and the first one within fast one at the same temperature *T*_X1_^fast^~203 K together with the second change within fast regime at *T*_X2_^fast^ = 295 K are observed. The glass-to-liquid transition at *T*_g_^DSC^ is marked by the vertical line. The regions A_slow_, B_slow,1_, B_slow,2_, B_fast,1_, B_fast,2_, C_fast,1_ and C_fast,2_, are fitted by the Arrhenius Equation or Vogel-Fulcher-Tammann-Hesse (VFTH) Equation with the fitting parameters listed in Table 1.

Figure 3 displays the time scale, *τ*_c_^slow^, *τ*_c_^fast^ of the corresponding slow and fast spectral components as a function of the inverse temperature 1/*T*. The three basic regions of the distinct mobility of the TEMPO probes in *cis*-1,4-PIP10k can be clearly distinguished and subsequently marked as A, B and C. The one-component broad spectra from slow spin probe motion occur in the low temperature region A_slow_ from 100 K to 150 K. On increase the temperature within region B, the bimodal spectra appear at *T*_X1_^slow^ = *T*_X,ini_^fast^ = 155 K and persist up to *T*_c_~250 K.

Moreover, in this region of the so-called dynamic heterogeneity of TEMPO mobility, two slow (B_slow,1_, B_slow,2_) and fast (B_fast,1_, B_fast,2_) sub-regions are observed at *T*_X2_^slow^ or *T*_X1_^fast^, respectively. Within this superposed region B, the sensitivity of both the slow- and fast-motion components to *T*_g_^DSC^ is evident compared to *cis*-1,4-PIP0.8k [39] as well as 1-PrOH [38] which exhibit only a phase change at *T*_g_ in the slow component. Note that the differences between polymeric *cis*-1,4-PIP10k and oligomeric *cis*-1,4-PIP0.8k will be compared in detail below in separate part of the Discussion section. Finally, the ESR spectra in region C simulated as the singlet narrow spectra reveal the occurrence of two fast sub-regimes at *T*_X2_^fast^ = 295 K.

The six slow and fast regions can be described by the Arrhenius equation *τ*_c,i_(*T*) = *τ*_∞,i_ exp[*E*_i_*/*RT*] with the pre-exponential factor, *τ*_∞,i_, the activation energy, *E*_i_* and the regression coefficient given in Table 1. However, for experimental data of the highest-T-region C_fast,2_, the Vogel-Fulcher-Tamman-Hesse (VFTH) equation, *τ*_c_(*T*) = *τ*_∞_[*B*^VFTH^/(*T* − *T*_0_^VFTH^)] [57,58,59], worked very well. Here, *τ*_∞_ is the pre-exponent, *B*^VFTH^ is the VFTH parameter and *T*_0_^VFTH^ is the divergence temperature. This formula gives more realistic description than the Arrhenius one that provides the unrealistically low pre-exponent 3.3 × 10^−17^ s.

**Figure 4:**
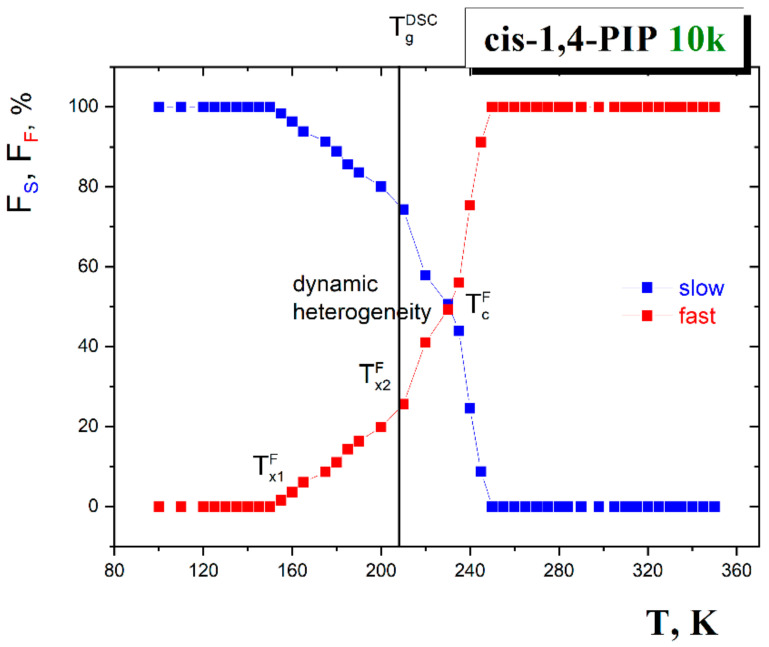
Fractions of the slow and fast reorienting spin probe TEMPO in *cis*-1,4-PIP10k as a function of temperature [40]. The crossover effects *T*_X1_^F^, *T*_X2_^F^ and *T*_c_^F^ are in agreement with the characteristic ESR temperatures of the correlation time *T*_X1_^slow^, *T*_X,in_^fast^~155 K and *T*_c_~250 K in Figure 3. The glass-to-liquid transition *T*_g_^DSC^ is depicted by the vertical line.

The temperature dependences of the relative fractions of the individual spectral components *F*_slow_ and *F*_fast_ are given in Figure 4. In general, they exhibit the changes at the characteristic ESR temperatures *T*_X1_^F^, *T*_X2_^F^ and *T*_c_^F^ which are consistent with the characteristic ESR temperatures from the τ_c_ vs. 1/*T* plot: *T*_X1_^slow^= *T*_x,ini_^fast^, *T*_X2_^slow^ = *T*_X1_^fast^ and *T*_c_.

### 3.2. PALS Data

**Figure 5:**
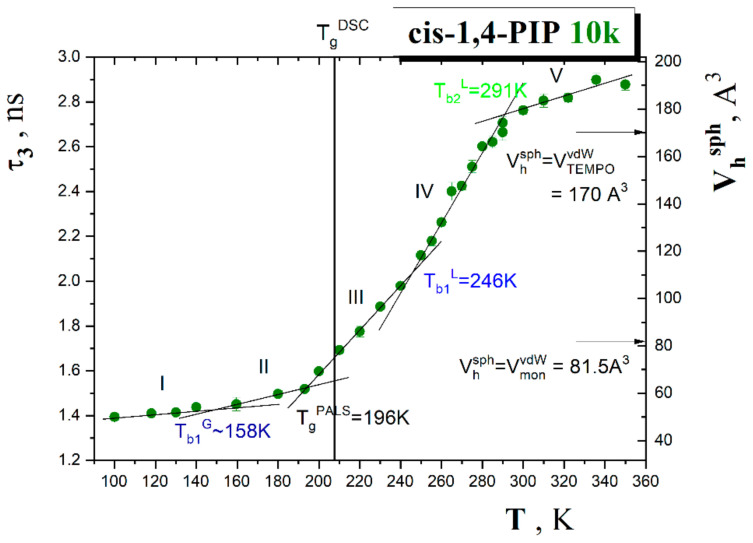
The *o*-Ps lifetime, *τ*_3_, and equivalent spherical-like free volume, *V*_h_^sph^, as a function of temperature in the pure *cis*-1,4-PIP10k medium with the characteristic PALS temperatures: *T*_b1_^G~^158 K, *T*_g_^PALS^ = 194 K, *T*_b1_^L^ = 246 K, *T*_b2_^L^ = 291 K determined as an intersction poin points of two linear fits. Error bars are included. The equivalent spherical free volumes *V*_h_^sph^ compared to the vdW volume of monomer *V*_mon_^vdW^ and vdW volume of TEMPO *V*_TEMPO_^vdW^ are marked by the corresponding arrows. The glass-to-liquid temperature *T*_g_^DSC^ = 208 K is depicted by the vertical line.

Figure 5 displays the temperature dependence of the mean *o*-Ps lifetime, *τ*_3_, in the pure *cis*-1,4-PIP10k sample in the temperature range from 100 K to 350 K. The PALS measurements were performed in slow heating mode with 5–10 K steps. The PALS response exhibits five regions of distinct thermal behavior of the *o*-Ps annihilation which are characterized by four characteristic PALS temperatures, i.e., *T*_b1_^G~^158 K, *T*_g_^PALS^~196 K, *T*_b1_^L^ = 246 K, *T*_b2_^L^ = 291 K. On the right axis of Figure 5, the mean equivalent free volume, *V*_h_ = (4π/3)*R_h_*^3^, determined by the standard quantum-mechanical model of *o*-Ps in a spherical hole, relates to the observed mean *o*-Ps lifetime, *τ*_3_, and the mean sphere size of free volume entity *R_h_* [23,24,43,44,45]:

(1) $$\tau_{3}=\tau_{3,0}{\left\{1-R_{h}/\left(R_{h}+\Delta R\right)+\left(1/2\pi \right)\sin\left[2\pi R_{h}/\left(R_{h}+\Delta R\right)\right]\right\}}^{-1}$$

where *τ*_3,0_ = 0.5 ns is the spin averaged lifetime of *p*-Ps and *o*-Ps and Δ*R* = *R*_0_ *− R_h_* = 1.66 Å is the thickness of the electron layer around the free volume hole which was obtained for well known cavities in molecular crystals and zeolites [43,44,45]. In general, in polymers as complex chain-like systems, one can expect rather the aspherical shape of the intersegmental space and its anisotropic thermal expansion as demonstrated for several model oligomeric and polymeric substances [46,47,48,49]. However, the equivalent free volume *V*_h_^sph^ = (4π/3)*R_h_*^3^ is commonly used as an approximate measure of the free volume hole size [23,24].

As in Figure 2 and Figure 3 the glass-to-liquid transition temperature, *T*_g_^DSC^, is also included. In the *τ*_3_-*T* plot, in the lower-*T* region, the first bend temperatures at *T*_b1_^G^~0.76*T*_g_^DSC^ or (~0.81 *T*_g_^PALS^) and the most pronounced effect are situated in the glassy state below *T*_g_^DSC^. This effect lying a bit lower than *T*_g_^DSC^ is designated as *T*_g_^PALS^. This shift of T_g_ is due to the different rates of the temperatures change during the DSC and PALS experiments.

In addition, in the liquid state above *T*_g_^PALS^ ≈ *T*_g_^DSC^, further two bend effects are observed at *T*_b1_^L^ = 1.18*T*_g_^DSC^ (1.26*T*_g_^PALS^) and *T*_b2_^L^ = 1.40*T*_g_^DSC^ (1.48*T*_g_^PALS^). Again as in the ESR case, the origins of these changes in the free volume expansion with increasing temperature remain to be revealed.

### 3.3. LS Data

#### 3.3.1. Phenomenological Analysis of the Slow Dynamics

**Figure 6:**
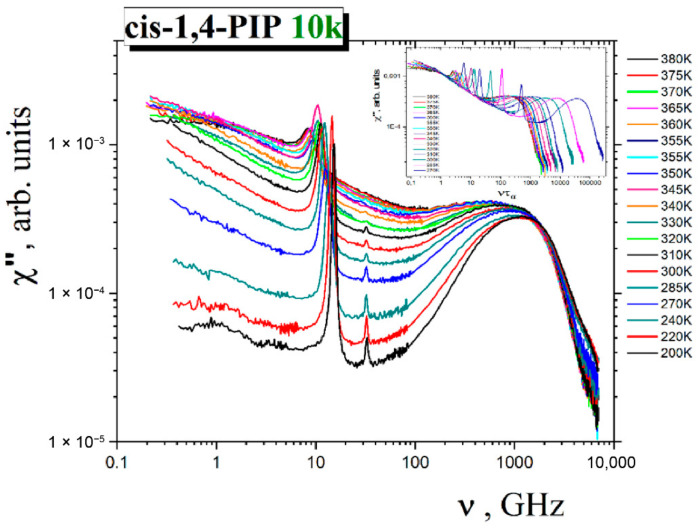
Light scattering spectra of *cis*-1,4-PIP10k at a series of temperatures from a broad temperature range: 200–380 K. The inset shows the master plot; for its construction see the text.

Figure 6 displays the susceptibility χ″(*ν*) = *I*(*ν*)/[*n*(*ν*)+1] of the pure *cis*-1,4 PIP10k sample in the temperature range from 200 K to 380 K over the frequency range 0.4–6 954 GHz. Here, *I*(*ν*) is the light scattering intensity and *n*(*ν*) = [exp(*hν*/*kT*)-1]^−1^ is the Bose temperature factor. In general, the LS spectra consist of three components (1) the power-law wing with negative slope at low frequencies, (2) the fast relaxation with apparent positive slope in the intermediate frequency range and finally, (3) the boson peak (BP) in the spectra density representation. According to the present knowledge state, the α relaxation stems from a large-scale cooperative interchain segmental relaxation of the oligomer chains. The smaller-scale fast dynamics can be attributed to localized motion between and within the segments of the polymeric chains and finally, the BP is caused by quasi-acoustical vibrations.

At the highest temperatures 370 K, 375 K and 380 K, the segmental α relaxation peak in the susceptibility spectra can be seen. In order to extract the α relaxation time,*τ_α_*, the susceptibility spectra at these temperatures were fitted by the Cole-Davidson (CD) function defined as [60]:

*χ*″(*ν*) = *A*Im {1/(1 + i2π*ντ*_CD_)*^β^*_CD_} (2)

where *ν* is the frequency, *A*, *τ*_CD_, and *β*_CD_ are fitting parameters characterizing the amplitude, the weight maximum position and the high frequency asymptotic power law behavior of the α relaxation peak, respectively. The characteristic α relaxation time *τ*_α_ was determined from the equation *τ*_α_ = *τ*_CD._*β*_CD_ [12,13,51,52,61].

In the susceptibility spectra at lower temperatures, the maximum of the structural α relaxation, *τ*_α_, was obtained from a master plot for the α relaxation peak. To construct this master plot, the susceptibility spectra at three temperatures 370 K, 375 K and 380 K as a function of the reduced frequency *ντ*_α_ were plotted. Next, the data for lower temperatures were added. For each temperature, a value of *τ*_α_ was found out to reach the best overlap of the high-frequency parts of the α relaxation peaks for different temperatures from 270 K to 380 K.

**Figure 7:**
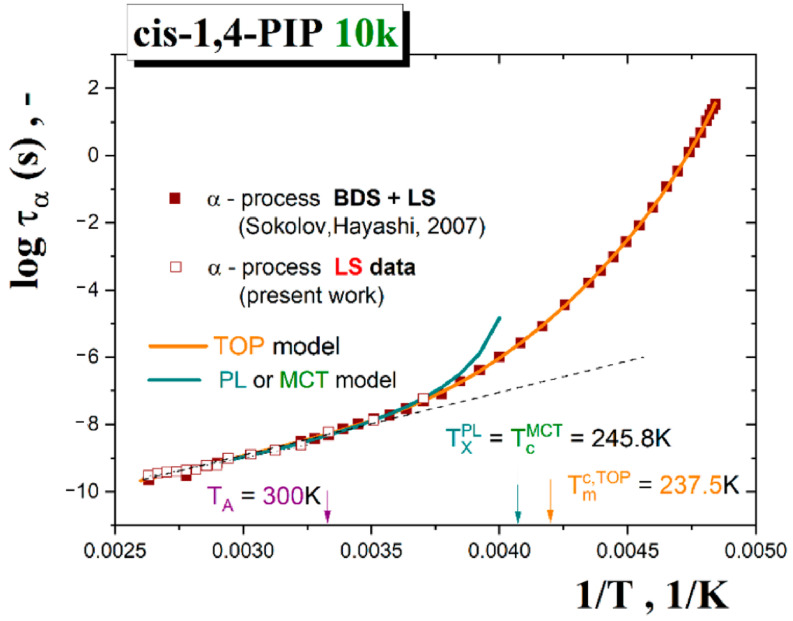
Arrhenius plot of the segmental α relaxation time, τ_α_, for *cis*-1,4-PIP10k as obtained from the present Light Scattering LS work together with those from a combined BDS and LS study of the same *cis*-1,4-PIP10k in Ref. [50]. Fit curves of the Power Law (PL) function or the Mode Coupling theory (MCT) model (Equation (10)) over intermediate- and high-*T* region as well as the TOP model (Equation (11)) over the whole *T* range are included and commented in the text in detail.

Figure 7 shows the obtained segmental α relaxation times as evaluated from the present LS measurements together with those from the BDS ones including three points from very restricted LS study [50].

#### 3.3.2. Analysis of the Fast Dynamics

In Figure 6, the wing of the LS spectra in *cis*-1,4-PIP10k can be described by a power law expression:

χ″_wing_(ν) = C/*ν* (3)

The fast motion relaxation on shorter time scales than the slow segmental α relaxation time is well described by the Gilroy-Phillips (GP) model of thermally activated jumps in asymmetric double-well potentials [62,63]. This GP model was used in the temperature range up to 310 K, where the wing is still dominating the relaxation at lower frequencies. This model assumes the exponential distribution of the barrier heights *V*, exp(−*V*/*V*_0_), with some typical barrier *V*_0_. The susceptibility has a low-frequency power-law tail with the slope b = *T*/*V*_0_ at *T* << *V*_0_ that goes to 1 at higher *T*. From the right side of the peak, the slope is as for the Debye relaxation, −1. For simplicity, we approximated this behavior by the function with correct asymptotes.

*χ*″_fast_ (*ν*) = D*(*ν*/*ν*_0_)*^b^*/(1+(*ν*/*ν*_0_)^2^)^(1+*b*)/2^ (4)

The boson peak (BP) in the spectral density can be well described by a universal log-normal function [64] which was used many times in fitting its spectral shape in various materials:

*χ*”_BP_(*ν*) = ν*A_BP_*exp(−(ln *ν*/*ν*_BP_)^2^/2σ^2^) (5)

Finally, the global fitting function used for the total light scattering spectrum is

*χ*″(*ν*) = C/ν*^a^* + D*(ν/ν_0_)*^b^*/(1+(*ν*/*ν*_0_)^2^)^(1+*b*)/2^ + ν*A_bp_*exp(−(ln *ν*/*ν*_bp_)^2^/2σ^2^) (6)

**Figure 8:**
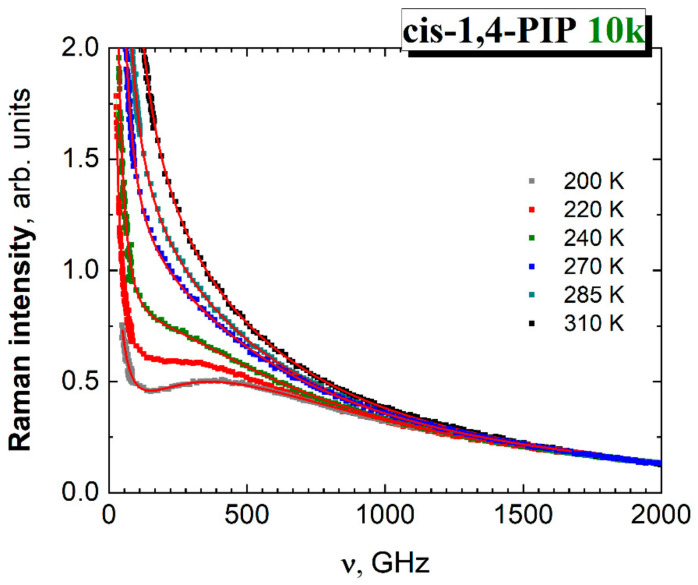
Raman spectra in spectral density representation from 200 K to 310 K. Fits using the additive model Equation (6) with parameters from Table 2 are included by red lines.

Figure 8 shows the resulting fits using the additive model (Equation (6)) over the temperature range from 200 K to 310 K.

**Figure 9:**
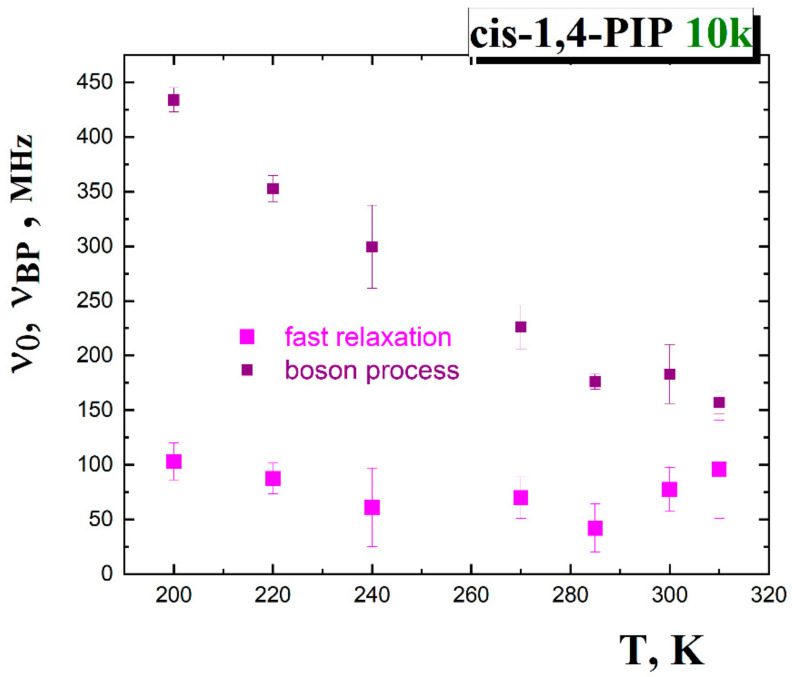
Temperature dependences of the fast motion and the boson process frequencies in *cis*-1,4-PIP10k.

In Figure 9, the boson peak frequency, *ν*_BP_, decreases with increasing temperature, while the relaxation frequency of fast motion maximum, *ν*_0_, is more or less constant within the error bars interval. However, it is difficult to separate the fraction of the fast relaxation motions at *T* > 310 K due to too smooth LS spectrum, many unknown parameters in the individual components and unknown shape of the right slope of the α relaxation that exceeds the wing at *T* > 310 K. At these high temperatures, the Mode Coupling theory (MCT) analysis appears to be more suitable approach.

#### 3.3.3. MCT Analysis

The idealized mode coupling theory (I-MCT) gives some prediction about the temperature evolution of the susceptibility minimum between the slow structural α process and the fast dynamics [65,66], which can be summarized as in the equation:

*χ*” = *χ*″_min_ {[b(*ν*/*ν*_min_)^a^ + a(*χ*″/*χ*″_min_)^−b^]/(a + b)} (7)

where *ν*_min_ and *χ″*_min_ are the frequency or amplitude of minimum, respectively. The exponents *a* and *b* describe the low-frequency part of the fast dynamics and the high frequency part of the segmental α process.

**Figure 10:**
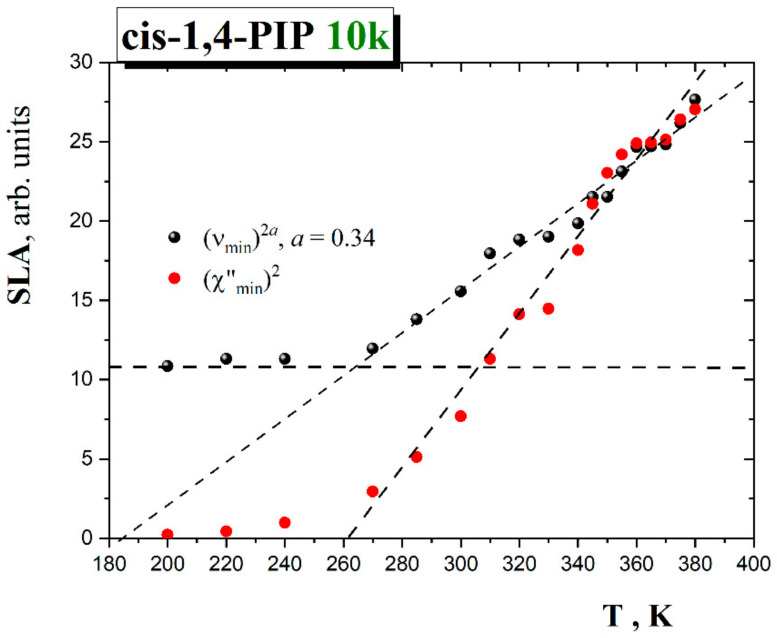
Scaled law amplitude (SLA) as a function of temperature for *cis*-1,4-PIP10k.

In Figure 10, the linearized scaling law amplitudes (SLA) provide the critical temperature of the MCT model:

*ν*_min_∝(*T*−*T*_c_^I-MCT^)^1/2a^ (8)

*χ*″_min_∝(*T*−*T*_c_^I-MCT^)^1/2^ (9)

The SLA for *ν*_min_ and *χ*″_min_ give the values for *T*_c_^I-MCT^ = 180 K or 262 K, respectively, by taking into account all the data range. In this case, a significant discrepancy between *T*_c_^I-MCT^ values as obtained from the analysis of *ν*_min_ and *χ″*_min_ exists.

For resolving this problem, the fact was took into account that even at low temperatures, a minimum in the susceptibility spectra (see Figure 6) formed by some additional relaxation with a bump near 1 GHz was seen, while within the MCT, a minimum near *T_g_* was not observed. In this case, the value (*ν*_min_)^2*0.34^ near 200 K as a low-temperature limit and to see the crossing of (*ν*_min_)^2*0.34^(*T*) with this value was taken into account. This way of analysis gives also *T*_c_^I-MCT^ ≈ 262 K (see Figure 10). So, from the analysis can be concluded that the analysis of the temperature dependence of the position and amplitude of the susceptibility minimum gives for *T*_c_^I-MCT^ ≈ 262 K. Note that the onset of the SLA enhancements starts already somewhat above 240 K.

#### 3.3.4. Segmental α Relaxation of *cis*-1,4-PIP10k in Terms of the Power Law (PL) Function or Mode Coupling Theory (MCT) Model and the Two-Order Parameter (TOP) Model 

The extracted time scales of the structural (segmental) relaxation as a function of temperature in Figure 7 can be described by various expressions, such as the power law (PL) function [67] or equivalently the afore-mentioned idealized mode coupling theory (I-MCT) model [65,66] and the two-order parameter (TOP) model [68,69,70]. Returning to Figure 7, both types of dynamic models of the segmental α relaxation time data of *cis*-1,4-PIP10k are tested. The PL function which is related to the prediction of the I-MCT are expressed by the following formula:

(10) $$\tau_{\alpha }\left(T\right)=\tau_{\infty ,\alpha }{\left[\left(T-T_{x}\right)/T_{x}\right]}^{-\mu }$$

where *τ*_∞,α_ is the pre-exponential factor, *T*_X_ is the characteristic temperature of PL function and MCT model and *μ* is the coefficient. Both are valid at rather intermediate and higher temperatures in relatively lower viscosity of materials [71,72]. In our case of *cis-1,4 PIP10k*, the relaxation data above *T* = 271 K (from the first LS point in Figure 7 up to the final 380 *K*) can be satisfactorily described by Equation (10) which provides the characteristic dynamic crossover temperature of *T*_x_^PL^ = *T*_c_^I-MCT^ = 245.8 K and μ = 3.1.

Alternative description and the related solid-like and liquid-like domain picture interpretation of the segmental dynamics in *cis*-1,4-PIP10k over the whole measured temperature range can be provided by the two-order parameter (TOP) model [68,69,70]. This model is based on the modified VFTH (M-VFTH) formula:

(11) $$\tau_{\alpha }\left(T\right)=\tau_{\alpha ,\infty }exp\left[E_{\tau }^{*}/RT\right]\exp\left[BF\left(T\right)/\left(T-T_{0}\right)\right]$$

where $\tau \left(T\right)$ is the structural relaxation time, $\tau_{\infty }$ is the pre-exponent factor, $E_{\tau }^{*}$ is the activation energy above $T_{m}^{*}\approx T_{A}$**,** $T_{0}$ is the divergence temperature, *B* is the coefficient and *F*(*T*)is a probability function for solid-like domains. The latter quantity is defined as:

(12) $$F(T)=1/\{\exp[\kappa (T-T_{m}^{c})]+1\}$$

where *κ* describes the sharpness of the probability function between solid-like and liquid-like domains and $T_{m}^{c}$ is the characteristic TOP temperature, i.e., the critical temperature where the free energy of a crystallizing liquid is equal to that of the crystal $\Delta G_{lq}=\Delta G_{cr}$ or, in the general case of non-crystallizing glass-formers, the free energy of a liquid is equal to that of a solid: $\Delta G_{lq}=\Delta G_{sol}$. In the present case, the parameters are as follows: $\log\tau_{\alpha ,\infty }=$ −14.3, $E_{\tau }^{*}=$ 37.7 J/mol, *B* = 525.1 K, *κ* = 0.054 1/K, $T_{m}^{c}=$ 237.5 K and $T_{0}=$ 169.7 K. Both the characteristic model temperatures *T*_X_^PL^ = *T*_c_^I-MCT^ and *T*_m_^c,TOP^ are rather close to each other and they will be discussed in a connection with the obtained ESR and PALS data.

## 4. Discussion

### 4.1. ESR vs. PALS Data Comparison in Terms of 2A_zz′_ vs. τ_3_ and τ_c_ vs. τ_3_

**Figure 11:**
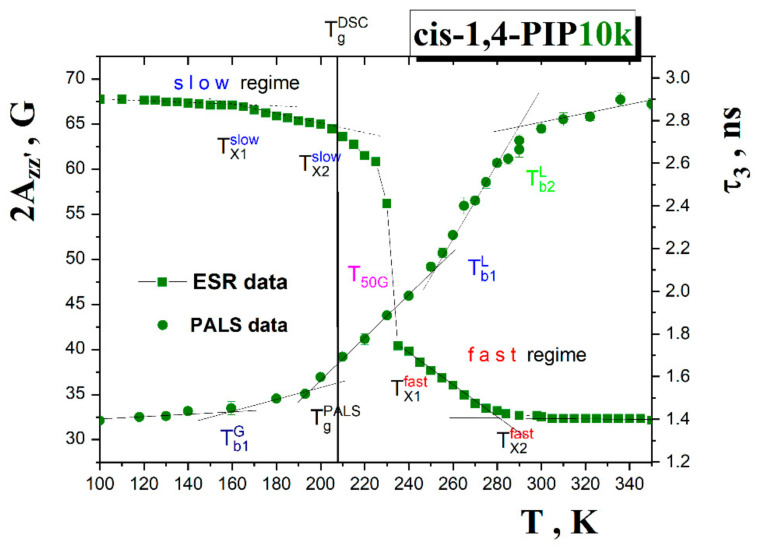
Comparison of spectral parameter of mobility, 2*A*_zz′_, with the *ortho*-positronium (*o*-Ps) lifetime, *τ*_3_, as measures of the spin probe TEMPO mobility or the local free volume, respectively.

First, the relationships between two independent extrinsic probe experiments, i.e., the data of the local dynamics of molecular spin probe TEMPO in *cis*-1,4-PIP10k at a level of the extrema line separation 2*A*_zz′_ and the free volume in the pure in *cis*-1,4-PIP10k as detected by *τ_3_* from Figure 2 and Figure 5 mutually compared in Figure 11 are discussed.

In the ESR response, the first weak decrease in 2*A*_zz′_ at *T*_X1_^slow,2Azz^~160 K lies close to the slight bend effect at *T*_b1_^G~^158 K in the *τ*_3_ vs. *T* plot within the glassy state of *cis*-1,4-PIP10k. As already mentioned above, in the Results section, the second decrease at *T*_X2_^slow,2Azz^~205 K lies in the vicinity of the glass-to-liquid temperature *T*_g_^DSC^ = 208 K. The most pronounced effect in the 2*A*_zz′_ vs. *T* dependence marking a transition of the spin probe TEMPO between its slow and fast motion regimes at conventionally defined *T*_50G_ = 232 K does not have any direct counterpart in the *τ*_3_-*T* plot, although it is not very distant from *T*_b1_^L^. Here, *τ*_3_(*T*_50G_)~2.07 ns and the corresponding equivalent spherical free volume size *V*_h_^sph^(*T*_50G_)~110 Å^3^ falls into the first empirical rule: *τ*_3_(*T*_50G_) = 2.17 ± 0.12 ns and *V*_h_^sph^(*T*_50G_) = 114 ± 15 Å^3^ [32]. On further increasing temperature in the supercooled liquid state of *cis*-1,4-PIP10k, the onset to the fast motion regime occurs at *T*_X1_^fast,2Azz~^240 K in 2*A*_zz′_-T plot in the vicinity of the mild bend effect in the PALS response at *T*_b1_^L^ = 246 K. Finally, in the normal liquid state of *cis*-1,4-PIP10k, the TEMPO probes exhibit further acceleration at roughly *T*_X2_^fast,2Azz~^282 K within the fast motion regime accompanied by the further narrowing of the triplet spectrum which is in plausible agreement with the onset to the quasi-plateau effect at *T*_b2_^L~^291 K. Here, *τ*_3_(*T*_X2_^fast^)~2.75 ns corresponding to the equivalent spherical free volume *V*_h_^sph^(*T*_X2_^fast^)~180 Å^3^ are in a good agreement with the second rule, i.e., *τ*_3_(*T*_Xi_^fast^) = 2.85 ± 0.15 ns and *V*_h_(*T*_Xi_^fast^) = 185±18 Å^3^ [32]. Thus, a mutual comparison of the changes in the spectral parameter of TEMPO probe 2*A*_zz′_ and those in the *o*-Ps lifetime in the pure *cis*-1,4-PIP10k medium revealed the following series of temperature coincidences, i.e., *T*_X1_^slow,2Azz^~*T*_b1_^G^, *T*_X2_^slow,2Azz^~*T*_g_^PALS^, *T*_X1_^fast,2Azz^~*T*_b1_^L^ and *T*_X2_^fast,2Azz~^*T*_b2_^L^.

**Figure 12:**
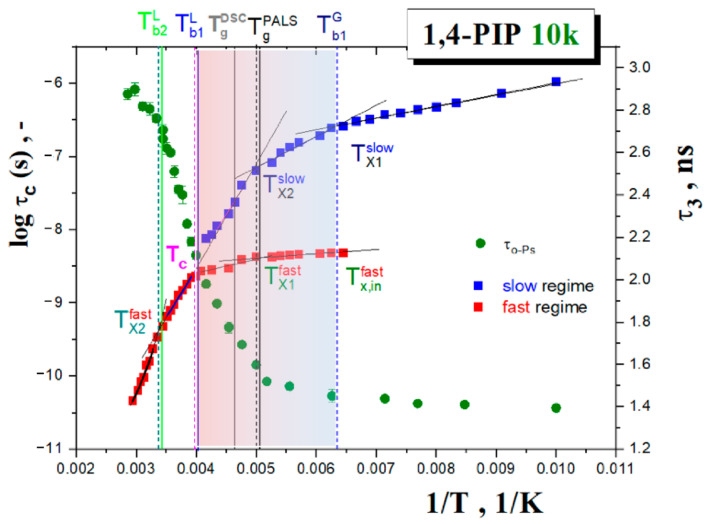
Inverse temperature dependences of time scales log *τ*_c_ and *τ*_3_ determined for the TEMPO reorientation or *o*-Ps probe annihilation behavior in *cis*-1,4-PIP10k, respectively [24].

Next, the ESR and PALS findings can be compared at a level of the respective ESR and PALS time scales from Figure 3 and Figure 5 as given in Figure 12.

In the low temperature region, an appearance of the fast component in the ESR spectrum is observed at *T*_X1_^slow^=*T*_X,in_^fast^ close to *T*_b1_^G^ at which a small increase in the *o*-Ps lifetime and related free volume expansion also occur. The second acceleration in the slow and simultaneously the slight first one in the fast component are found at *T*_x2_^slow^ and *T*_x1_^fast^ which lie in the vicinity of the *T*_g_^PALS^ and *T*_g_^DSC^ values.The full disappearance of the slow component at *T*_c_ = 250 K can be related to *T*_b1_^L^ = 246 K. Finally, the crossover effect between two fast motion sub-regimes at *T*_X2_^fast^ is in a good accord with an onset to the quasi-plateau effect in the normal liquid state around *T*_b2_^L^. The observed mutual PALS and ESR coincidences at a level of the changes of the respective time scales can be summarized as follows: *T*_X1_^slow^ = *T*_X,in_^fast^ = *T*_b1_^G^, *T*_X2_^slow^ = *T*_X1_^fast^ ≅ *T*_g_^PALS^ ≈ *T*_g_^DSC^, *T*_c_ ≅ *T*_b1_^L^ and finally, *T*_X2_^fast^ ≅ *T*_b2_^L^.

The mutual temperature coincidences of the various effects in the ESR and PALS responses indicate that the changes in the free volume expansion at the characteristic PALS temperatures are closely related to the changes in the dynamic state of the small molecular probe TEMPO. This strongly suggests the common physical origin of these mutually coinciding changes. The natural question arises: What physical processes are behind the various crossover effects in the atomic probe annihilation and the molecular mobility in *cis*-1,4-PIP10k?

**Figure 13:**
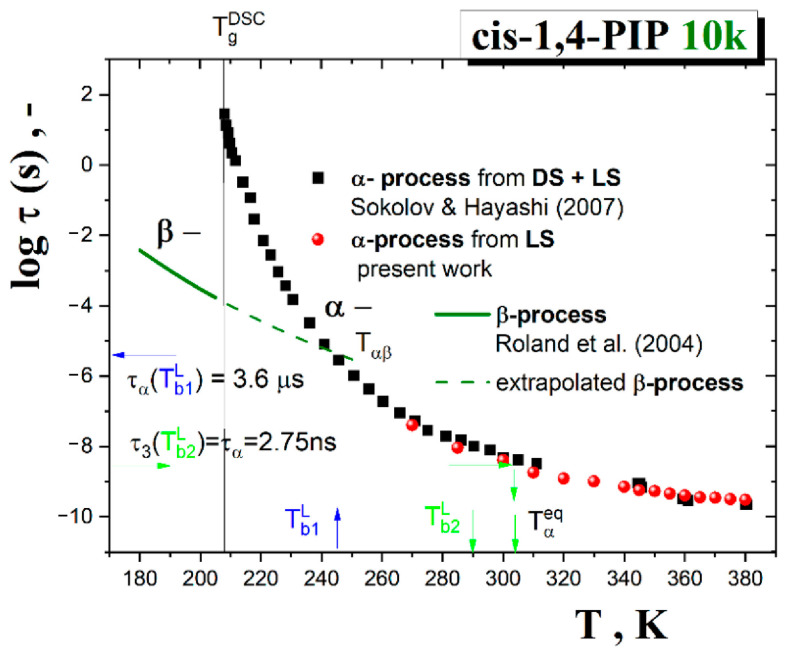
Time scales of *o*-Ps annihilation, *τ*_3_, and the primary α process, *τ*_α_, from the present LS and published BDS [50] data and the secondary β process, *τ*_β_, from DS study on 1,4-PIP500k in Ref. [73].

In Figure 13, the relationships between the mean *o*-Ps lifetimes at the characteristic PALS temperature in the liquid state and the mean characteristic segmental α relaxation times, *τ*_α_, and the mean secondary relaxation, *τ*_β_ are showed [68,69,70]. In the former case it can be seen that the so-called equivalent α temperature, *T*_α,eq_, i.e., the temperature at which the PALS time scale equals to LS and BDS [33,34,35,36,37,38], being 303 K is not too distant from the second characteristic PALS temperature, *T*_b2_^L^ = 291 K. This plausible match of *T* findings suggests that an onset of the quasi-plateau effect in the PALS response is related to the segmental α dynamics of *cis*-1,4-PIP10k sample. Moreover, this is fully consistent with the generally accepted bubble concept of the *o*-Ps annihilation in low viscous organic media [73].

Next, based on the finding of the relation *T*_X2_^fast^~*T*_b2_^L^, the second acceleration in the TEMPO reorientation within the fast motion regime is at least influenced by the segmental process. As for the first characteristic PALS temperature at *T*_b1_^L^, being close to the *T*_c_ from Figure 3, the situation is a bit more complicated. At *T*_b1_^L^, the mean time scale of the segmental α relaxation reaches a few μs, i.e., about three orders of magnitude longer than the *o*-Ps lifetime *τ*_3_(*T*_b1_^L^) = 2.1 ns. On the other hand, after a commonly used linear extrapolation of the secondary β scale [74] into the liquid state, one can estimate the so-called αβ merging temperature, *T*_αβ_ ≈ 245 K, at which both the relaxation processes should merge and further continue as a unified αβ process. This mutual temperature coincidence *T*_c~_*T*_b1_^L^~*T*_αβ_ seems to suggest that the first slight change in the free volume expansion between deeply and slightly supercooled liquid state as well as the appearance of the pure fast reorientation regime of the TEMPO molecules could be related to the occurrence of the potentially unified αβ process in *cis*-1,4-PIP10k.

**Figure 14:**
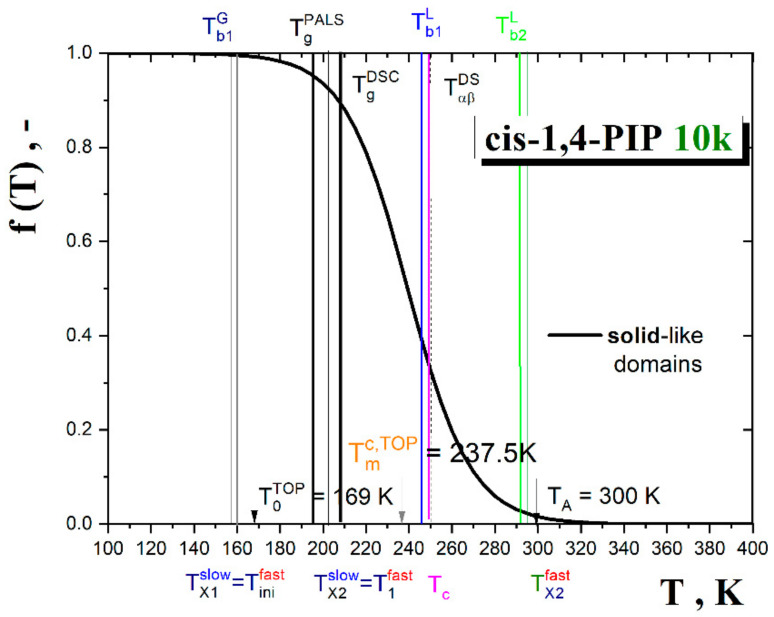
Probability function of solid-like domains, *f*(*T*), or the liquid-like regions, 1 − *f*(*T*), obtained from Equation (11) fitting the segmental α relaxation time by Equation (11) with the characteristic PALS and ESR (from spectral simulations) temperatures together with the characteristic TOP temperatures as well as with the glass-to-liquid temperature *T*_g_^DSC^ and the αβ merging temperature *T*_αβ_^DS^ [74].

Next, these mutual phenomenological relationships between the characteristic PALS and ESR temperatures will be further discussed in the context of both models applied for the segmental dynamics in Section 3.3.4. As presented in Figure 7, the MCT model equivalent to the empirical PL function and the TOP model provide satisfactory fits with the corresponding model temperatures: *T*_X_^PL^ = *T*_c_^I-MCT^ = 245.8 K or *T*_m_^c,TOP^ = 237.5 K, respectively. These quite close values are in plausible agreement with the characteristic PALS and ESR temperatures *T*_c~_*T*_b1_^L^. Thus, both models offer the corresponding interpretations in terms of the apparent divergence of the density fluctuation or the crossover between the solid-like and liquid-like domains. In the former case, the unphysical divergence might be removed by adding of the hopping term in the extended version of the mode coupling theory (E-MCT) that provides the same *T*_c_^E-MCT^ as its idealized version *T*_c_^I-MCT^ [63]. On the other hand, Figure 14 represents the probability function of the solid-like domains extracted from Equation (11) of the TOP model together with all the characteristic PALS and ESR temperatures as well as the afore-mentioned merging temperature *T*_αβ_ from DS study [74].

The quite satisfactory closeness between the characteristic PALS and ESR temperatures in the glassy state with an onset of the liquid-like domains at around *T*_0_^TOP^ was observed. Further, as given above, the first characteristic ESR and PALS temperatures in the supercooled liquid state lie in the vicinity of the crossover temperature where the liquid-like domains begin to dominate over the solid-like ones, i.e., in the vicinity of a transition between the strongly supercooled to the weakly supercooled liquid. Finally, the high-*T* characteristic PALS and ESR temperatures *T*_b2_^L^ and *T*_X2_^fast^ appear close to the third characteristic TOP temperature, namely, the Arrhenius one *T*_A_ = 300 K. Here, the liquid-like domains are substantially predominant over the solid-like ones and *cis*-1,4-PIP10k behaves as a normal low viscosity liquid.

### 4.2. Comparison of ESR and PALS Responses for Polymeric cis-1,4-PIP10k vs. Oligomeric cis-1,4-PIP0.8k

**Figure 15:**
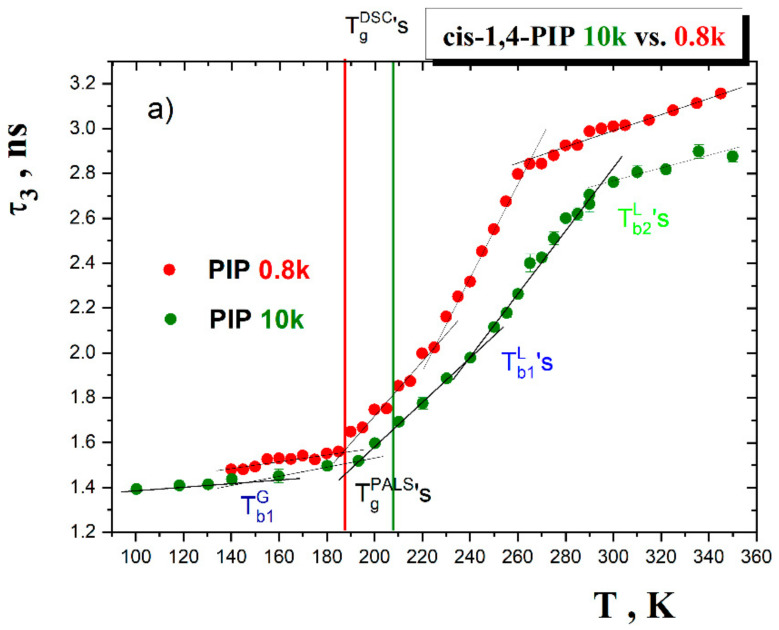

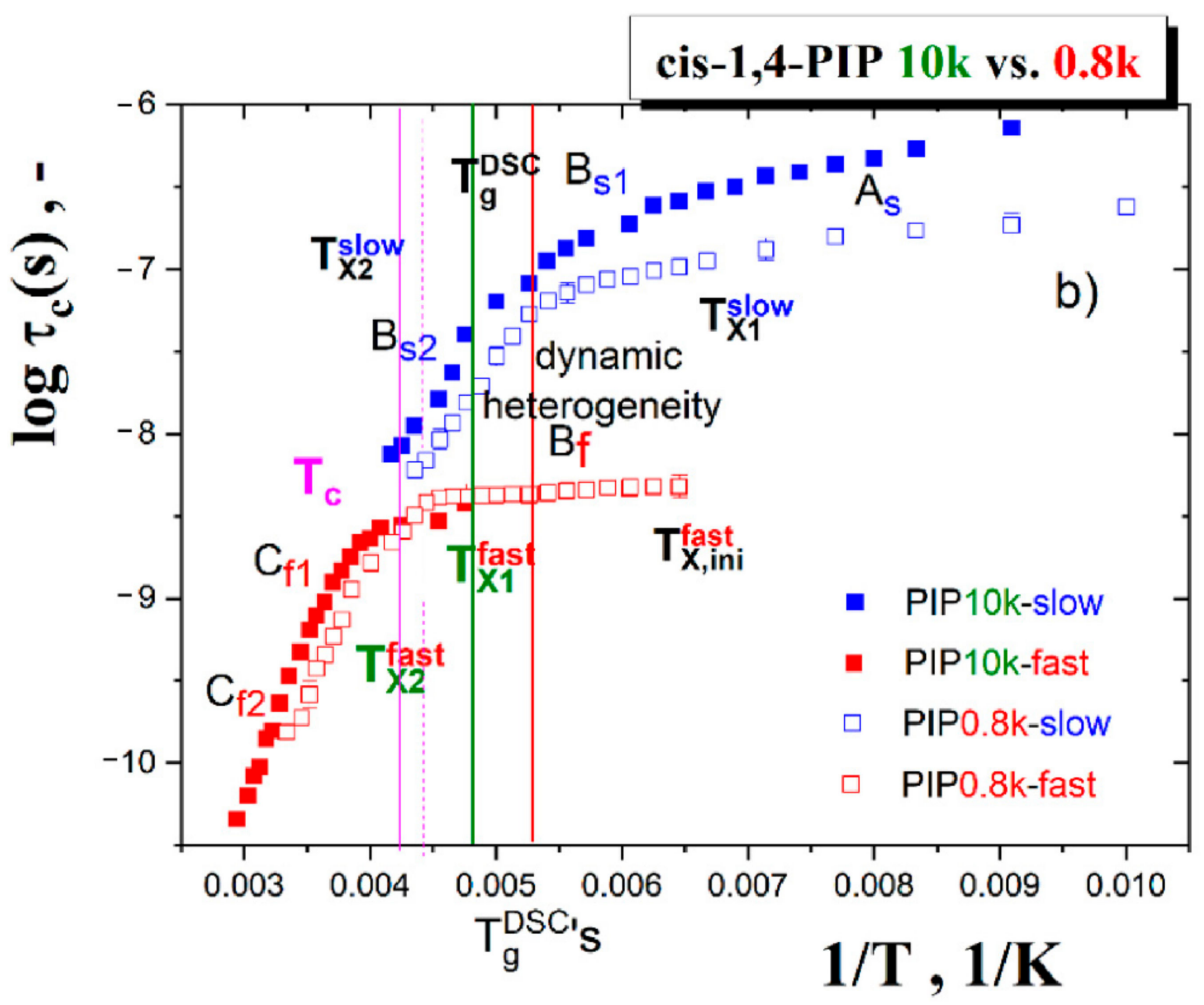
Comparison of the ESR and PALS data for *cis*-1,4-PIP10k vs. *cis*-1,4-PIP0.8k [39] in terms of (**a**) *τ*_3_ vs. *T* and (**b**) log *τ*_c_ vs.1/*T* plots.

It is of interest to compare the present PALS and ESR data on polymeric *cis*-1,4-PIP10k with its oligomeric *cis*-1,4-PIP0.8k counterpart [39] and to reveal the size effect of the corresponding chains on both free volume microstructure and spin probe TEMPO dynamics. The former glass former contains about 140 basic structural units~[*CH_2_-CH=C(CH_3_)-CH_2_*]~per chain, while the later one is essentially shorter with ca. only 12 monomers/chain. Figure 15a,b demonstrate that with the increasing chain length and thus, the increasing strength of inter- and intra-segmental interactions, the free volume reduces and consequently, the molecular reorientation dynamics slows down. The difference in the TEMPO mobility, as expressed by the respective correlation times, appears to be larger within the slow motion regime in the glassy state as well as above *T*_g_^DSC^ in the strongly supercooled liquid one, where the free volume difference is not so large. On the other hand, in the slightly supercooled liquid state, this free volume difference becomes larger up to the corresponding *T*_b2_^L^, while the difference in the rotation dynamics of TEMPO within the fast motion region is smaller. At higher temperatures, i.e., in the quasi-plateau region above *T*_b2_^L^, due to the well-known artefact nature of the *o*-Ps response in low viscosity media mentioned above, such a comparison cannot be performed. The observed trends in the spin probe TEMPO dynamics could be ascribed to the different degrees of the local perturbation of the respective oligomeric or polymeric medium by the molecular probe TEMPO. According to Figure 5, the temperature dependence of the mean equivalent spherical free volume *V*_h_^sph^ estimated from Equation (1) is also included. We can see that the *V*_h_^sph^ values of *cis*-1,4-PIP10k are smaller than the vdW volume of the spin probe TEMPO up to ca. 280 K. Consequently, the slow dynamics of TEMPO in the glassy and deeply supercooled liquid state of *cis*-1,4-PIP10k is slower than that in *cis*-1,4-PIP0.8k due to somewhat tighter surroundings around the TEMPO molecules. In both the *cis*-1,4-PIP glassformers the mean *o*-Ps lifetime at the corresponding transition temperatures *T*_c_’s,*τ*_3_(*T*_c_), reaches the same value around 2.1 ns corresponding to the *V*_h_^sph^(T_c_) ≅ 115 Å^3^, being smaller than *V*_TEMPO_^vdW^ = 170 Å^3^. It seems to imply a local deformation of the immediate surroundings of the TEMPO molecules in *cis*-1,4-PIP/TEMPO system in comparison to the pure bulk *cis*-1,4-PIP medium. On further increase of the temperature, this local perturbation of the respective medium should be weaken which results in the closer rotation dynamics of TEMPO within the fast motion regime among both the *cis*-1,4-PIP media. This hypothesis is further discussed in the next sub-section.

### 4.3. Relationships between the Time Scales from ESR and BDS, LS and Further Dynamic Techniques

**Figure 16:**
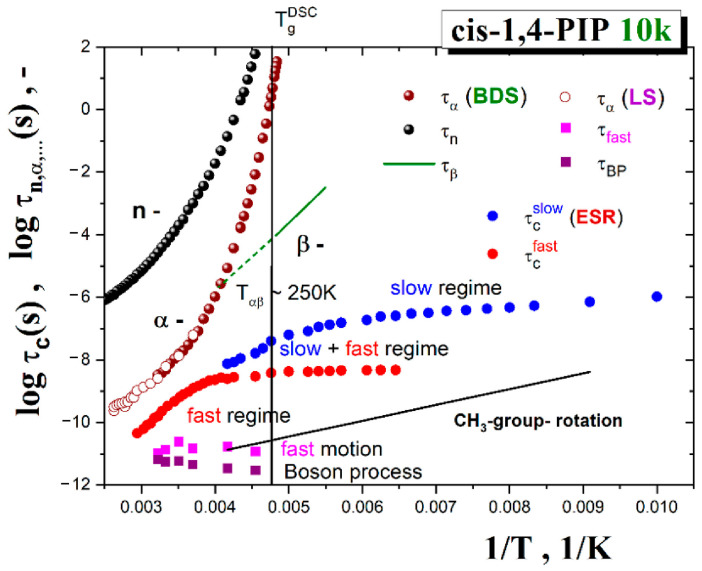
Relaxation map of *cis*-1,4-PIP10k sample consisting of BDS, light scattering (LS) and neutron scattering (NS) time scales together with those for the spin probe TEMPO correlation times in *cis*-1,4-PIP10k, *τ_c_*, obtained in the temperature range 100 K up to 400 K. Dynamic data include the chain relaxation time, *τ*_n_, from BDS [50], the segmental relaxation, *τ*_α_, from BDS [50] and LS and the secondary β relaxation, *τ*_β_, in 1,4-PIP500k over 180 K–205 K from BDS [74], the fast motion relaxation, τ_fast_, and boson peak, τ_boson_, from LS as well as CH_3_-jump rotation over 110–240 K from NS [75].

The local deformation concept of the molecular probe in organic medium, outlined in the previous sub-section, implies that the TEMPO molecule responds to some local dynamics in its immediate surroundings which should be similar or different to the local dynamics of the bulk medium. This would correspond to the full coupled or the decoupled situations, respectively. As already mentioned, in the case small molecular glass formers with the spin probe TEMPO in the fast motion regime, the former situation is observed due to very tight coupling of the spin probe dynamics and the structural relaxation of small molecules that are more compactly arranged around TEMPO [37,38]. On the other hand, in chain media such as *cis*-1,4-PIP0.8k, the local structural situation is not so favorable partly due to the connectivity aspect of the medium constituents which do not allow compacted microstructure of the spin probe surroundings [39]. In this connection, the time scales of the spin probe TEMPO reorientation with those of numerous motional modes in *cis*-1,4-PIP10k were suitable to compare. Figure 16 presents the correlation time of TEMPO probe and the characteristic time scales of all the known six motional modes in *cis*-1,4-PIP10k detected by BDS [50,74] and NS [75] techniques as well as LS over a wide temperature range from 180 K to 380 K. These include relatively slow motional modes, such as the normal [50] and the primary α relaxations [50] from BDS and the present LS study. The secondary β relaxation [74] and relatively fast ones, such as the fast motion and the boson process, reveal LS investigation as well as the methyl group jump rotation [75]. By comparison with the ESR data, in contrast to the small molecular glass formers [37,38], the TEMPO time scales for polymeric *cis*-1,4-PIP10k in all the three, i.e., slow-, co-existing slow- and fast- and fast motion regimes between 0.75*T*_g_ and 1.15*T*_g_ do not follow any of the time scales of molecular motions. Indeed, they lie in between the relatively slow segmental α relaxation and the local fast motion dynamics. Thus, by considering the afore-mentioned PALS finding about the local deformation of the medium by the applied molecular probe, leading the different local potential field around it, this causes the modified local dynamics of the surrounding molecules of medium around the reporter’s molecular probe governing its rotation reorientation.

## 5. Conclusions

The rotation dynamics of small spin probe 2,2,6,6-tetramethylpiperidinyl-1-oxyl (TEMPO) in amorphous polymeric glass former, namely, *cis*-1,4-poly(isoprene) (*cis*-1,4-PIP10k) was investigated over a wide temperature range from 100 K to 360 K by means of electron spin resonance (ESR). Several regions of distinct spectral and related dynamic behavior of TEMPO were revealed via two parameters of spin probe mobility, i.e., the extrema separation of the spectra and the correlation time. A set of the changes in both parameters, characterized by ESR temperatures, were consistent with the free volume changes in the pure *cis*-1,4-PIP10k sample detected by PALS technique.

In order to identify the physical process responsible for the spin probe dynamics in the fast regime, the detailed dynamic study of *cis*-1,4-PIP10k medium was carried out by light scattering techniques focusing on the high-frequency relaxations of the medium constituents. Finally, a comparison of the time scales of both slow and fast motion regimes of TEMPO in *cis*-1,4-PIP10k with the found six motional modes in *cis*-1,4-PIP10k*,* i.e., a series of slow and fast relaxation modes from LS and BDS as well as NS techniques, strongly suggests the controlling factor of the spin probe mobility over a wide temperature region which consists in the local dynamics of the modified local surrounding of the molecular probe TEMPO.

## Figures and Tables

**Table 1:** The Arrhenius and the Vogel-Fulcher-Tamman-Hesse (VFTH) equations parameters of the spin probe TEMPO dynamics in *cis*-1,4-PIP10k. B_i_^VFTH^ = 503.3 ± 49.6; *T*_0,i_^VFTH^ = 216.7 ± 49.5 K, r = 0.989.

Region	Δ*T*, K	*τ*_∞,i_, s	*E*_i_*, kJ/mol	*r*
A_slow_	100–150	(2.52 ± 0.94) × 10^−8^	3.1 ± 0.1	0.998
B_slow,1_	155–200	(5.53 ± 0.65) × 10^−10^	8.1 ± 0.6	0.958
B_slow,2_	210–240	(2.06 ± 0.33) × 10^−14^	25.1 ± 2.1	0.973
B_fast,1_	155–200	(2.55 ± 0.93) × 10^−9^	0.8 ± 0.1	0.958
B_fast,2_	210–245	(2.99 ± 0.63) × 10^−10^	4.3 ± 0.7	0.944
C_fast,1_	255–284	(1.58 ± 0.65) × 10^−14^	25.2 ± 1.0	0.990
C_fast,2_	305–340	(7.67 ± 0.18) × 10^−13^		

**Table 2:** Spectral Parameters of the Fit by Equation (6).

*T*, K	*C*	*a*	*D*	*b*	*ν*_0_, GHz	*A*_BP_	*ν*_BP_, GHz	*σ*	δ^2^ = S_fast_/S_BP_
200	54	0.2	25	1	103	0.44	434	1.38	0.052
220	76	0.2	22.7	1	87.4	0.49	353	1.5	0.044
240	132	0.22	18.7	1	61	0.56	299	1.55	0.035
270	297	0.3	29.7	1	69.8	0.69	226	1.68	0.049
285	490	0.39	54.6	1	42	0.83	176	1.8	0.081
300	655	0.37	46.7	1	77.3	0.79	183	1.76	0.075
310	916	0.39	25.4	1	96	0.89	157	1.78	0.041

## Data Availability

Not applicable.

## References

[1] Angell C.A., Ngai K.L., McKenna G.B., McMillan P.F., Martin S.W. (2000). Relaxation in glassforming liquids and amorphous solids. J. Appl. Phys..

[2] Ediger M.D., Harrowell P. (2012). Perspective: Supercooled liquids and glasses. J. Chem. Phys..

[3] Stillinger F.H., Debenedetti P.G. (2013). Glass transition thermodynamics and kinetics. Annu. Rev. Condens. Matter Phys..

[4] Biroli G., Garrahan J.P. (2013). Perspective: The glass transition. J. Chem. Phys..

[5] Frick B.D., Richter D., Ritter C. (1989). Structural changes near the glass transition-neutron diffraction on a simple polymer. Europhys. Lett..

[6] Eckstein E., Qian J., Hentschke R., Thurn-Albrecht T., Steffen W., Fischer E.W. (2000). X-ray scattering study and molecular simulation of glass forming liquids: Propylene carbonate and salol. J. Chem. Phys..

[7] Colmenero J., Arbe A. (2013). Recent progress on polymer dynamics by neutron scattering: From simple polymers to complex materials. J. Polym. Sci. B Polym. Phys..

[8] Richter D., Monkenbusch M., Arbe A., Colmenero J. (2005). Neutron Spin Echo in Polymer Systems.

[9] Zorn R. (2003). Microscopic dynamics of glass-forming polymers. J. Phys. Condens. Matter.

[10] Berne B.J., Pecora R. (1976). Dynamic Light Scattering.

[11] Kruk D., Herrmann A., Rössler E.A. (2012). Field-cycling NMR relaxometry of viscous liquids and polymers. Prog. Nucl. Magn. Reson. Spectrosc..

[12] Lunkenheimer P., Köhler M., Kastner S., Loidl A., Wolynes P.G., Lubchenko V. (2012). Dielectric Spectroscopy of Glassy Dynamics. Structural Glasses and Supercooled Liquids: Theory, Experiment, and Applications.

[13] Kremer F., Schönhals A. (2003). Broadband Dielectric Spectroscopy.

[14] Barlow A.J., Lamb J., Matheson A.J. (1966). Viscous behavior of supercooled liquids. Proc. Roy. Soc..

[15] Schönhals A., Kremer F., Hofmann A., Fischer E.W., Schlösser E. (1993). Anomalies in the scaling of the dielectric α-relaxation. Phys. Rev. Lett..

[16] Stickel F., Fischer E.W., Richert R.J. (1995). Dynamics of glass-forming liquids. I. Temperature-derivative analysis of dielectric relaxation data. J. Chem. Phys..

[17] Schönhals A. (2001). Evidence for a universal crossover behavior of the dynamic glass transition. Europhys. Lett..

[18] Alegría A., Colmenero J., Mari P.O., Campbell I.A. (1999). Dielectric investigation of the temperature dependence of the nonexponentiality of the dynamics of polymer melts. Phys. Rev..

[19] Leon C., Ngai K.L. (1999). Rapidity of the change of the Kohlrausch exponent of the α-relaxation of glass-forming liquids at T_B_ or T_β_ and consequences. J. Phys. Chem..

[20] Ngai K.L., Roland M. (2002). Development of cooperativity in the local segmental dynamics of poly(vinylacetate): Synergy of thermodynamics and intermolecular coupling. Polymer.

[21] Berthier L., Biroli G., Bouchaud J.-P., Cipelletti L., van Saarloos W. (2011). Dynamical Heterogeneities in Glasses, Colloids and Granular Materials.

[22] Hutchison C., Bhattarai A., Wang A., Mohanty U. (2019). Fluctuation Effects in the Adam–Gibbs Model of Cooperative Relaxation. J. Phys. Chem. B.

[23] Dlubek G., Siedel A. (2008). Encyklopedia of Polymer Science and Technology.

[24] Jean Y.C., Mallon P.E., Schrader D.M. (2003). Principles and Application of Positron and Positronium Chemistry.

[25] Veksli Z., Andreis M., Rakvin B. (2000). ESR spectroscopy for the study of polymer heterogeneity. Progr. Polym. Sci..

[26] Cameron G.G., Booth C., Price C. (1989). Comprehensive Polymer Science.

[27] Berliner L.J. (1976). Spin Labelling Theory and Applications.

[28] Bartoš J., Šauša O., Bandžuch P., Zrubcová J., Krištiak J. (2002). Free volume factor on supercooled liquid dynamics. J. Non-Cryst. Solids.

[29] Švajdlenková H., Bartoš J. (2009). Spin probe mobility in relation to free volume and relaxation dynamics of glass-formers: A series of spin probes in poly(isobutylene). J. Polym. Sci. Polym. Phys..

[30] Rabold G.P. (1969). Spin-probe studies. II. Applications to polymer characterization. J. Polym. Sci..

[31] Bartoš J., Andreozzi L., Faetti M., Šauša O., Račko D., Krištiak J. (2006). Free volume in poly(propylene glycol) and its relationships to spin probe reorientation. J. Non-Cryst. Solids.

[32] Švajdlenková H., Šauša O., Iskrová-Miklošovičová M., Majerník V., Krištiak J., Bartoš J. (2012). On the relationships between guest molecular dynamics and free volume in a series of small molecular and polymer glass-formers. Chem. Phys. Lett..

[33] Bartoš J., Šauša O., Krištiak J., Blochowicz T., Rössler E. (2001). Free-volume microstructure of glycerol and its supercooled liquid-state dynamics. J. Phys. Cond. Matter.

[34] Bartoš J., Šauša O., Račko D., Krištiak J., Fontanella J.J. (2005). Positron annihilation lifetime response and relaxation dynamics in glycerol. J. Non-Cryst. Solids.

[35] Bartoš J., Šauša O., Köhler M., Švajdlenková H., Lunkenheimer P., Krištiak J., Loidl A. (2011). Positron annihilation and broadband dielectric spectroscopy: A series of propylene glycols. J. Non-Cryst. Solids.

[36] Bartoš J., Šauša O., Cangialosi D., Alegria A., Švajdlenková H., Krištiak J., Arbe A., Colmenero J. (2012). Positron annihilation and relaxation dynamics from dielectric spectroscopy: Poly (vinylmethylether). J. Phys. Cond. Matter.

[37] Bartoš J., Švajdlenková H. (2017). On the mutual relationships between spin probe mobility, free volume and relaxation dynamics in organic glass-formers: Glycerol. Chem. Phys. Letts..

[38] Bartoš J., Švajdlenková H., Šauša O., Lukešová M., Ehlers D., Michl M., Lunkenheimer P., Loidl A. (2016). Molecular probe dynamics and free volume in organic glass-formers and their relationships to structural relaxation: 1-propanol. J. Phys. Cond. Matter.

[39] Švajdlenková H., Arrese-Igor S., Nógellová Z., Alegría A., Bartoš J. (2017). Molecular dynamic heterogeneity in relation to free volume and relaxation dynamics in organic glass-formers: Oligomeric cis-1,4-poly(isoprene). Phys. Chem. Chem. Phys..

[40] Švajdlenková H., Šauša O., Čechová K., Bartoš J. (2018). Dynamic and free volum aspects of spin probe TEMPO in organic glass formers: Polymer cis-1,4-poly(isoprene). AIP Conf. Proc..

[41] Budil D.E., Lee S., Saxena A.S., Freed J.H. (1996). Nonlinear-least-squares analysis of slow-motion EPR spectra in one and two dimensions using a modified Levenberg-Marquardt algorithm. J. Magn. Reson..

[42] Kansy J. (1996). Microcomputer program for analysis of positron annihilation lifetime spectra. Nucl. Inst. and Methods Phys. Res. A.

[43] Tao S.J. (1972). Positronium annihilation in molecular substrances. J. Chem. Phys..

[44] Eldrup M., Lightbody D., Sherwood J.N. (1981). The temperature dependence of positron lifetimes in solid pivalic acid. Chem. Phys..

[45] Nakanishi H., Jean Y.C., Wang S.J., Sharma S.C. (1988). Positron Annihilation Studies of Fluids.

[46] Consolati G. (2002). Positronium trapping in small voids: Influence of their shape on positron annihilation results. J. Chem. Phys..

[47] Consolati G. (2005). On the thermal expansion of nanohole free volume in perfluoropolyethers. J. Phys. Chem..

[48] Consolati G. (2006). Temperature dependence of holes free volume in polypropylene glycols. Appl. Phys. Letts..

[49] Bradac C., Consolati G., Quasso S. (2009). Temperature dependence of free volume in atactic polypropylene. Eur. Polym. J..

[50] Sokolov A.P., Hayashi Y. (2007). Breakdown of time-temperature superposition: From experiment to the coupling model and beyond. J. Non-Cryst. Solids.

[51] Havriliak S., Negami S. (1967). A complex plane representation of dielectric and mechanical relaxation processes in some polymers. Polymer.

[52] Havriliak S., Negami S. (1966). A complex plane analysis of α-dispersions in some polymer systems. J. Polym. Sci. C Polym. Symp..

[53] Surovtsev N.V. (2017). Suppression of spurious background in low-frequency Raman spectroscopy. Optoelectron. Instrum. Data Process..

[54] Surovtsev N.V., Wiedersich J.A.H., Novikov V.N., Rössler E., Sokolov A.P. (1998). Light-scattering spectra of fast relaxation in glasses. Phys. Rev..

[55] Barshilia H.C., Li G., Shen G.Q., Cummins H.Z. (1999). Depolarized light scattering spectroscopy of Ca_0.4_K_0.6_(NO_3_)_1.4_: A reexmination of the “knee”. Phys. Rev. E.

[56] Popova V.A., Surovtsev S.V. (2014). Transition from Arrhenius to non-Arrhenius temperature dependence of structural relaxation time in glass-forming liquids: Continuous versus discontinuous scenario. Phys. Rev..

[57] Vogel H. (1921). Das Temperaturabhaengigkeitsgesetz der Viskositaet von Fluessigkeiten. Phys. Z..

[58] Fulcher G.S. (1925). Analysis of recent measurements of the viscosity of glasses. J. Am. Ceram. Soc..

[59] Tamman G., Hesse W. (1926). Die abhängigkeit der viscosität von der temperatur bie unterkühlten Flüssigkeiten. Z. Anorg. Allg. Chem..

[60] Davidson D.W., Cole R.H. (1951). Dielectric relaxation in glycerol, propylene glycol, and n-propanol. J. Chem. Phys..

[61] Böttcher C.J.F., Bordewijk P. (1978). Theory of Electric Polarization.

[62] Gilroy K.S., Phillips W.A. (1981). An asymmetric double-well potential model for structural relaxation processes in amorphous materials. Philos. Mag. B.

[63] Adichtchev S.V., Surovtsev N.V., Wiedersich J., Brodin A., Novikov V.N., Rössler E.A. (2007). Fast relaxation processes in glasses as revealed by depolarized light scattering. J. Non-Cryst. Solids.

[64] Malinovsky V.K., Novikov V.N., Sokolov A.P. (1991). Log-normal spectrum of low-energy vibrational excitations in glasses. Phys. Lett..

[65] Götze W. (1999). Recent tests of the mode-coupling theory for glassy dynamics. J. Phys. Cond. Matter.

[66] Götze W., Sjögren L. (1992). Relaxation processes in supercooled liquids. Rep. Progr. Phys..

[67] Taborek T., Kleinman R.N., Bishop D.J. (1986). Power-law behavior in the viscosity of supercooled liquids. Phys. Rev..

[68] Tanaka H. (2005). Two-order-parameter model of the liquid-glass transition. I. relation between glass transition and crystallization. J. Non-Cryst. Solids.

[69] Tanaka H. (2005). Two-order-parameter model of the liquid-glass transition. II. structural relaxation and dynamic heterogeneity. J. Non-Cryst. Solids.

[70] Tanaka H. (2005). Two-order-parameter model of the liquid-glass transition. III. Universal patterns of relaxations in glass-forming liquids. J. Non-Cryst. Solids.

[71] Mallamace F., Bra C., Corsaro C., Leone N., Spooren J., Chen S.H., Stanley H.E. (2010). Transport properties of glass-forming liquids suggest that dynamic crossover temperature is as important as the glass transition temperature. Proc. Natl. Acad. Sci. USA.

[72] Mallamace F., Corsaro C., Stanley H.E., Chen S.H. (2011). The Role of the Dynamical Crossover Temperature and the Arrest in Glass Forming Fluids. Eur. Phys. J. E.

[73] Winberg P., Eldrup M., Maurer F.H. (2012). Free volume dilatation in polymers by ortho-positronium. J. Chem. Phys..

[74] Roland C.M., Schroeder M.J., Fontanella J.J., Ngai K. (2004). Evolution of the dynamics in 1,4-polyisoprene from a nearly constant loss to a Johari-Goldstein β-relaxation to the α-relaxation. Macromolecules.

[75] Zorn R., Frick B., Fetters L.J. (2002). Quasielastic neutron scattering study of the methyl group dynamics in polyisoprene. J. Chem. Phys..

